# Vascular transcriptome profiling identifies *Sphingosine kinase 1* as a modulator of angiotensin II-induced vascular dysfunction

**DOI:** 10.1038/srep44131

**Published:** 2017-03-09

**Authors:** Mateusz Siedlinski, Ryszard Nosalski, Piotr Szczepaniak, Agnieszka H. Ludwig-Gałęzowska, Tomasz Mikołajczyk, Magdalena Filip, Grzegorz Osmenda, Grzegorz Wilk, Michał Nowak, Paweł Wołkow, Tomasz J. Guzik

**Affiliations:** 1 Department of Internal and Agricultural Medicine, Faculty of Medicine, Jagiellonian University Medical College, Kraków, Poland; 2 British Heart Foundation Centre for Excellence, Institute of Cardiovascular and Medical Sciences, University of Glasgow, Glasgow, Scotland, UK; 3 Centre for Medical Genomics-OMICRON, Jagiellonian University Medical College, Kraków, Poland

## Abstract

Vascular dysfunction is an important phenomenon in hypertension. We hypothesized that angiotensin II (AngII) affects transcriptome in the vasculature in a region-specific manner, which may help to identify genes related to vascular dysfunction in AngII-induced hypertension. Mesenteric artery and aortic transcriptome was profiled using Illumina WG-6v2.0 chip in control and AngII infused (490 ng/kg/min) hypertensive mice. Gene set enrichment and leading edge analyses identified *Sphingosine kinase 1 (Sphk1)* in the highest number of pathways affected by AngII. Sphk1 mRNA, protein and activity were up-regulated in the hypertensive vasculature. Chronic sphingosine-1-phosphate (S1P) infusion resulted in a development of significantly increased vasoconstriction and endothelial dysfunction. AngII-induced hypertension was blunted in *Sphk1*^−/−^ mice (systolic BP 167 ± 4.2 vs. 180 ± 3.3 mmHg, p < 0.05), which was associated with decreased aortic and mesenteric vasoconstriction in hypertensive *Sphk1*^−/−^ mice. Pharmacological inhibition of S1P synthesis reduced vasoconstriction of mesenteric arteries. While Sphk1 is important in mediating vasoconstriction in hypertension, *Sphk1*^−/−^ mice were characterized by enhanced endothelial dysfunction, suggesting a local protective role of Sphk1 in the endothelium. S1P serum level in humans was correlated with endothelial function (arterial tonometry). Thus, vascular transcriptome analysis shows that S1P pathway is critical in the regulation of vascular function in AngII-induced hypertension, although *Sphk1* may have opposing roles in the regulation of vasoconstriction and endothelium-dependent vasorelaxation.

Hypertension is a pathogenetically complex disorder affecting more than 1 billion people worldwide^1^. Etiology of the disease remains unknown in the majority of adult patients. It is widely acknowledged that function of the vascular system, kidney and sympathetic nervous system is critical for regulating blood pressure (BP) levels^1^. Since the existence of genetic predisposition to develop abnormally high BP is well known, significant efforts have been made in order to identify particular genes affecting hypertension using genome-wide association studies (GWAS)^2^,^3^,^4^,^5^. However, clinical translation of such studies is in the early phase and requires detailed characterization of the effect of certain genes and molecular pathways on the function of key organs involved in BP regulation.

High throughput techniques significantly contributed to unraveling mechanisms of complex disorders and phenotypes such as type II diabetes^6^, chronic obstructive pulmonary disease^7^ or hypertension and BP regulation^2^,^3^,^4^,^5^ in humans. All the above studies focused on the association between genetic polymorphisms and disease status using Mendelian randomization concept allowing establishing causal relationships. Complexity of the pathogenesis of hypertension makes uncovering key drivers of the disease difficult in humans. Models of the disease such as angiotensin II (Ang II) infusion or DOCA salt hypertension, represent most common causes of primary hypertension in humans and allowed to unravel the important mechanisms of hypertension and associated target organ damage. Systemic vascular resistance is critical for the physiological regulation of blood pressure. At the same time, hypertension promotes development of vascular dysfunction and atherosclerosis in the larger vessels. Importantly, while vascular oxidative stress and inflammation have been clearly linked to hypertension and its consequences^8^,^9^, the effects of chronic Ang II infusion on distinct vascular beds is yet to be addressed in a systematic manner.

Vascular dysfunction may have divergent mechanisms and consequences in various vascular beds. In the aorta, it may represent vascular target organ damage but may also contribute to vascular stiffening important for hypertension development. In mesenteric arteries, which represent typical resistance vessels, increased vascular tone may directly translate into increased blood pressure.

Investigation of transcriptome creates an opportunity for gaining a unique insight into potential vascular mechanisms of Ang II-dependent hypertension. Transcriptome profiling studies *ex vivo* have so far focused on kidneys following 2 days Ang II infusion^10^, heart in the model of Ang II–induced cardiac fibrosis^11^,^12^ or abdominal aortic aneurysm in ApoE knockout mice^13^. Despite the difficulties with obtaining cell-type specific and homogenous RNA pool, all the above studies clearly showed that Ang II displays systemic effects on transcriptome and important information on the disease process studied could be obtained using this high-throughput technique.

The aim of current study was to profile the transcriptome of key vessels of normotensive and hypertensive mice using Ang II-induced hypertension model and further to identify genes and biological pathways that may causally affect development of hypertension *in vivo*. Both thoracic and abdominal regions of the aorta, selected in the current study, are characterized by hypertrophy following Ang II treatment *in vivo*^14^, yet they show different expression pattern of Ang II receptor subtypes AT1a and AT1b^15^,^16^. This may confer differential roles of these areas of the aorta in hypertension^15^,^16^, thus investigation and characterization of these two regions is a key to the understanding mechanism of Ang II action in an *in vivo* model. Superior mesenteric vascular bed, which is another part of the vasculature selected for transcriptome profiling, is a part of the vascular resistance system, critical for blood flow regulation^17^,^18^. Since mesenteric arteries express AT1 receptors and are susceptible to Ang II-mediated dysfunction *in vivo*^19^,^20^, investigation of similarities and differences in gene expression in these three areas of vasculature may provide important molecular information on specific changes occurring during Ang II-mediated hypertension *in vivo*.

## Results

### Transcriptome analysis

**Figure 1:**
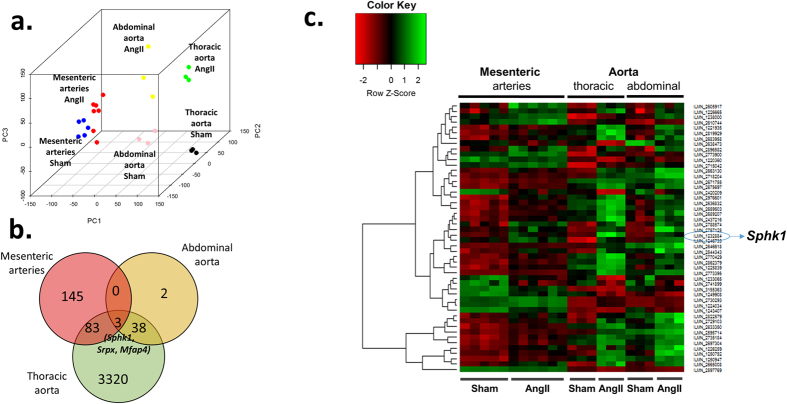
Principal component plot comprising all expressed RNA transcripts (**a**), differentially expressed RNA transcripts in all 3 tissue types (**b**) and heatmap of top 50 RNA transcripts affected by Ang II *in vivo* (**c**). (**a**) Top 3 principal components were calculated using expression intensities, normalized using robust spline normalization method, of approximately 16 000 RNA transcripts detected (detection p-value < 0.01) in all samples; (**b**) Three genes that expression is significantly changed in all 3 tissue types by Ang II infusion are depicted on Venn diagram, showing number of genes significantly affected by Ang II infusion *in vivo* in all 3 tissue types studied; (**c**) Heatmap depicts RNA transcripts most significantly affected by Ang II *in vivo* irrespectively of tissue type. *Srpx* = sushi-repeat containing protein, X-linked; *Sphk1/2* = Sphingosine kinase 1/2*; Mfap4* = microfibrillar-associated protein 4; PC = principal component.

Approximately 16,000 (out of 45,281) RNA transcripts were detected (detection p value < 0.01) in studied vascular tissues i.e. thoracic aorta, abdominal aorta and mesenteric arteries. Principal component analysis comprising RNA transcripts detected in all tissues provided a clear separation of samples according to tissue type and treatment status i.e. hypertensive vs. normotensive (Fig. 1a). Out of all transcripts expressed, 21.5% were differentially expressed (FDR corrected p < 0.05) in thoracic aorta between hypertensive and normotensive mice, while such proportion was lower for abdominal aorta (0.3%) or mesenteric arteries (1.4%). Among differentially expressed genes 46%, 91% and 67% were overexpressed in hypertensive as compared to normotensive mice in the thoracic aorta, abdominal aorta and mesenteric arteries respectively.

Among top 200 RNA transcripts associated with Ang II-induced hypertension in either of the examined tissues, 3 genes were previously associated with BP in GWA cohort studies. Expression of both subunits of the soluble guanylate cyclase (*Gucy1a3* and *Gucy1b3*) was significantly downregulated in both aortic compartments, but not in mesenteric arteries, in hypertensive mice as compared to control, while *Cyclin M2 (Cnnm2*) expression was significantly upregulated in mesenteric arteries of hypertensive mice as compared to control. None of the above genes showed a similar pattern of expression change following Ang II infusion in all vascular compartments studied.

Gene set enrichment analysis identified 68 gene sets significantly enriched in hypertension in thoracic aorta (see Supplementary Table 1 for top 20 sets). Subsequent leading edge analysis performed on these sets identified genes present in the highest number of pathways upregulated in Ang II-induced hypertension (Table 1). Among them, only *Sphk1* showed more than a 2-fold induction of expression in thoracic aorta of hypertensive mice as compared to control (Table 1).

Similar gene set enrichment analysis identified no pathways significantly enriched in hypertension in mesenteric arteries at FDR p value < 0.05. Therefore, leading edge analysis was based on 36 gene sets (see Supplementary Table 1 for top 20 sets) with normalized enrichment score >1.4 (nominal p value < 0.05) in mesenteric arteries. This analysis also identified *Sphk1* as one of the members of the highest number of gene sets enriched in mesenteric arteries following Ang II infusion (Table 1). Importantly, *Sphk1* gene was also among the 3 genes that changed in response to Ang II in all three vascular beds and among genes affected by Ang II infusion globally i.e. irrespectively of tissue type (Fig. 1b,c).

### *Sphk1* expression and activity in vasculature

**Figure 2:**
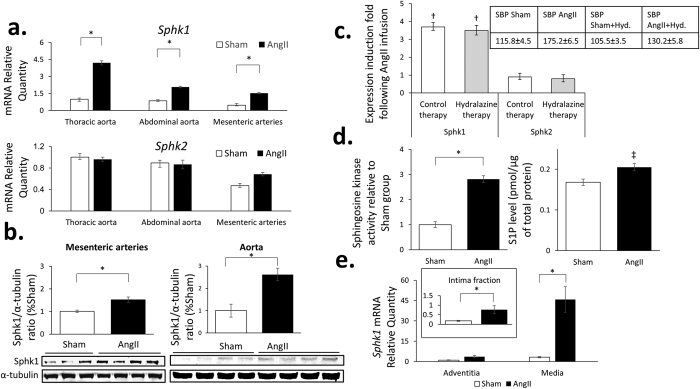
Sphk activity and expression of *Sphk* isoenzymes in the vasculature. (**a**) Expression of *Sphk1* and *Sphk2* mRNA analyzed by real-time RT-PCR normalized to the housekeeping *Tbp* gene in vascular compartments studied. *Sphk1/Sphk2* mRNA quantity is depicted relative to the expression observed in the thoracic aorta of Sham group (N = 6/group); (**b**) Sphk1 protein expression, studied by Western blotting in 2 independent experiments, in mesenteric arteries and aortic homogenates, that was normalized to α-tubulin n = 3–4/group. Detected bands and densitometric analyses are shown. Full-length blots are presented in Supplementary Figure 1; (**c**) Hydralazine (5 mg/kg/day) co-treatment effects on *Sphk1* and *Sphk2* mRNA expression in the thoracic aorta. Insert indicates changes of blood pressure upon Ang II and concomitant Ang II – Hydralazine treatment. n = 6/group; (**d**) Sphk activity (left) and S1P level (right) in thoracic aorta was quantified using ATP-depletion assay or competitive ELISA test respectively and results were normalized to a total protein content using Bradford reagent, n = 4–6/group; (**e**) Three layers of thoracic aorta were analysed in the context of *Sphk1* expression as depicted in the Methods section (n = 4/group). Results were normalized to the relative *Sphk1* expression in the adventitia of Sham group. *p < 0.05; ^†^p < 0.05 for *Sphk1* expression induction in AngII group vs. Sham; ^‡^p = 0.056 vs, Sham group; Tbp = TATA box binding protein; Sphk1/Sphk2 = Sphingosine kinase 1/2; Hyd = hydralazine.

Subsequent real-time PCR confirmed the above findings and showed a significant induction of *Sphk1* mRNA expression in all vascular compartments studied, while no significant effects of Ang II on *Sphk2* expression *in vivo* were observed (Fig. 2a). These increases translated into a significant increase in protein levels as exemplified by Western blotting (Fig. 2b and Supplementary Figure 1a,b). We observed a significant, yet modest, 1.8x increase in *Sphk1* mRNA expression in hearts of Ang II-infused mice as compared to control. This induction could not be confirmed using Western Blot analysis (Supplementary Figure 1b,c).

Moreover, induction of vascular *Sphk1* mRNA by Ang II infusion persisted when blood pressure raise was reduced by 76% by administration of hydralazine (Fig. 2c), indicating that increases observed were not a simple result of blood pressure raise but were likely caused by direct actions of Ang II *in vivo*.

The above observations were accompanied by a significant, 2.8x increase in sphingosine kinase activity as well as moderate increment in sphingosine-1-phosphate (S1P) concentration (Fig. 2d) in aortic homogenates of AngII-infused mice as compared to Sham control respectively.

An independent experiment focusing on different layers of thoracic aorta showed that Ang II effects on *Sphk1* expression *in vivo* were more pronounced in the media layer (Induction Fold (IF) = 14.2, p < 0.05) as compared to adventitia layer (IF = 3.6, p = 0.077) or endothelial cell -enriched fraction representing intima (IF = 4.5, p < 0.05, Fig. 2e). In baseline conditions expression of *Sphk1* was 3.2x and 18.1x higher in the media layer as compared to adventitia or intima respectively (Fig. 2e).

### Effects of chronic S1P infusion on vascular function *in vivo*

**Figure 3:**
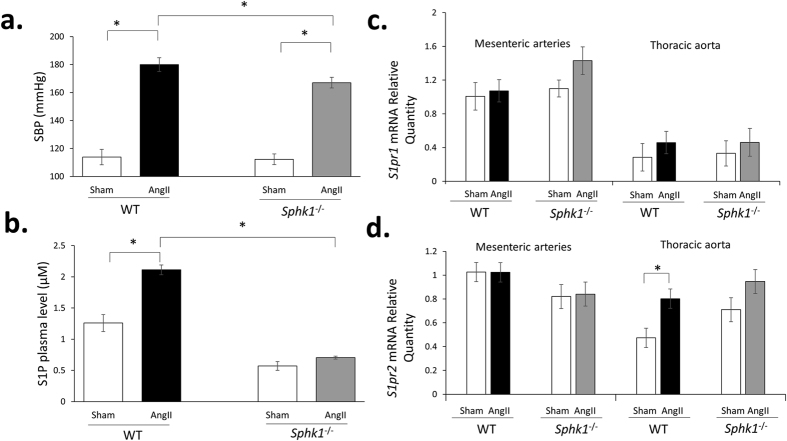
Systolic blood pressure level, plasma S1P level and expression of S1P receptors after 2 weeks of Ang II infusion in *Sphk1*^−/−^ and WT mice *in vivo.* (**a**) Average SBP after 2 weeks of infusion of AngII (490 ng/kg/min) is depicted. Mice underwent non-invasive blood pressure measurement by tail-cuff plethysmography (Visitech BP 2000 BP Analysis System), following a period of training before commencement of the treatment protocol, N = 5–6 for sham groups and n = 10 for AngII groups; (**b**) S1P level was quantified in plasma using competitive ELISA kit according to the manufacturer’s instructions (Echelon Biosciences, USA), n = 6/group; (**c**,**d**) Expression of *S1pr1 and S1pr2* was quantified in thoracic aorta and mesenteric arteries of normotensive and hypertensive WT and *Sphk1*^−/−^ mice and was normalized to the expression of *Tbp. S1pr1* and *S1pr2* mRNA quantity is depicted relative to the expression observed in the mesenteric arteries of Sham WT group (n = 6–8/group). Expression of the *S1pr3* receptor was not detected (Ct > 39 for all samples). *p < 0.05. Tbp = TATA box binding protein; S1pr1/S1pr2 = Sphingosine-1-Phosphate receptor 1/2; SBP = systolic blood pressure; S1P = sphingosine-1-phosphate.

AngII-induced hypertension was accompanied by a significant increase in plasma S1P level in wild-type (WT) mice (Fig. 3a,b). Therefore we studied the effects of chronic S1P delivery in normotensive mice *in vivo*. Chronic S1P infusion resulted in a 35% increment in plasma S1P level that was accompanied by a moderate, yet not significant increase in SBP by 10 mmHg *in vivo* (p = 0.35). S1P infusion resulted in significantly elevated degree of endothelial dysfunction and vascular contractility in mesenteric arteries (Supplementary Figure 2a–c).

### *Sphk1* deficiency and hypertension development *in vivo*

There was no significant difference in baseline blood pressure between *Sphk1*^−/−^ and WT mice (Fig. 3a). However, upon infusion of Ang II, *Sphk1*^−/−^ mice developed significantly less severe Ang II-induced hypertension *in vivo* as compared to WT mice (Fig. 3a). Importantly, no significant difference in heart rate was observed at both baseline conditions and upon Ang II infusion between WT and *Sphk1*^−/−^ mice (data not shown). Following 14-days AngII infusion period *Sphk1*^−/−^ mice developed less severe cardiac hypertrophy as compared to WT mice (Heart to body weight ratio for Ang II vs. Sham groups: 5.9 ± 0.22 mg/g vs. 5.5 ± 0.15 mg/g (p = 0.15) in *Sphk1*^−/−^ mice and 6.8 ± 0.21 mg/g vs. 5.8 ± 0.21 mg/g (p < 0.05) in WT mice). This was confirmed by an analysis of cardiomyocyte size (Supplementary Figure 3a,b). Further analyses revealed that effects of Ang II on fibrotic changes in the heart as well as IMT of cardiac arteries were similar in WT and *Sphk1*^−/−^ mice (Supplementary Figure 3c,d).

### Effects of AngII and *Sphk1* deficiency on S1P level and expression of S1P receptors *S1pr1* and *S1pr2*

*Sphk1*^−/−^ mice were characterized by 2–3x lower S1P plasma level as compared to WT mice (Fig. 3b) and approximately 30% ± 3% (p < 0.05) lower Sphk activity in the aorta. No significant compensatory effect of *Sphk1* deletion on *Sphk2* expression in vasculature was observed (data not shown). No significant effects of Ang II infusion or *Sphk1* deletion on *S1pr1* expression were observed (Fig. 3c). However, expression of this receptor was significantly higher in mesenteric arteries as compared to the thoracic aorta in both WT and *Sphk1*^−/−^ mice (Fig. 3c). We observed a significant induction of *S1pr2* expression in hypertensive WT mice as compared to normotensive WT mice in thoracic aorta (Fig. 3d). A similar trend (p = 0.07) regarding effect of AngII infusion was present in *Sphk1*^−/−^ mice as well (Fig. 3d). Similarly, to *S1pr1*, expression of *S1pr2* was significantly higher in mesenteric arteries as compared to the thoracic aorta in WT mice (Fig. 3d). Expression of *S1pr3* in the aorta could not be detected in vessels studied.

### Effects of *Sphk1* deficiency and pharmacological modulation on vascular contractility.

**Figure 4:**
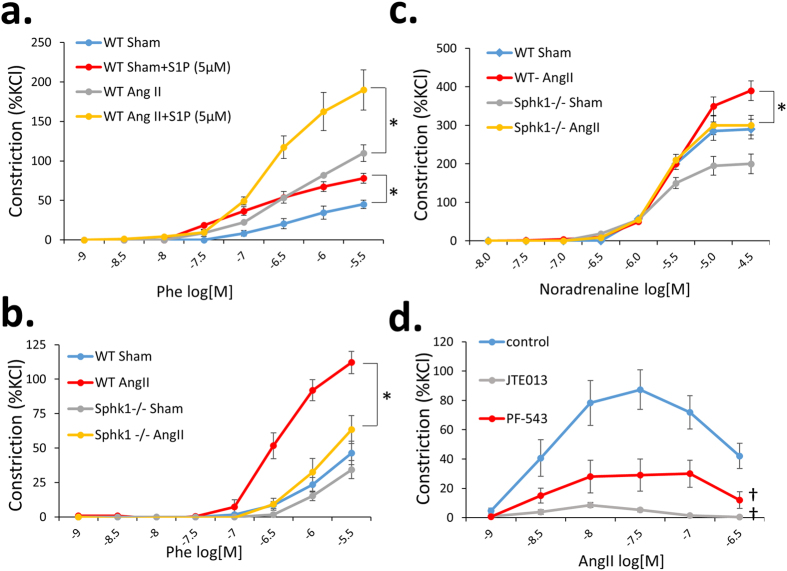
Contractile responses of the aorta and mesenteric arteries of WT and *Sphk1*^−/−^ mice and effects of pharmacological inhibition of S1P pathway on vessel contraction. (**a**) Tissue organ bath was performed using 2 mm aortic rings from WT mice preincubated for 5 min. with 5 μM S1P or with control buffer (n = 4/group); (**b**,**c**) Tissue organ bath was performed using 2 mm aortic rings (**b**) or 2^nd^ order mesenteric arteries (**c**) from WT and *Sphk1*^−/−^ mice with assessment of contractile force in response to Phe normalized to an initial KCl-induced contraction, n = 6/group; (**d**) Pharmacological studies on the effects of pre-incubation with 5 μM JTE-013 or 100 nM PF-543 on the contraction of WT Sham mesenteric arteries were performed using tissue organ bath, n = 4/group; *p < 0.05; ^†^p < 0.05 as compared to control group. Phe = phenylephrine; Ang II = angiotensin II; [M] = molar concentration.

Pre-incubation of WT thoracic aorta with S1P significantly increased vasoconstriction of aortic rings in response to phenylephrine (Phe) (Fig. 4a). This effect was also observed in mesenteric arteries and was also evident in vessels isolated from *Sphk1*^−/−^ mice (data not shown). On the contrary, *Sphk1* knockout led to a blunted increases in aortic and mesenteric arteries contractility to Phe and noradrenaline (respectively) in Ang II – hypertensive mice (Fig. 4b,c). In order to address this further, we studied the effects of S1P pathway modulators on vascular function *ex vivo*. Pre-incubation of mesenteric arteries with either Sphk1 inhibitor PF-543 or S1PR2 antagonist (JTE-013), significantly reduced Ang II-induced contraction *ex vivo* (Fig. 4d). Further tests performed on aortic rings showed that these inhibitory effects of JTE-013 on vessel contraction were dose-dependent and significant at a concentration of 100 nM (Supplementary Figure 4a).

### Effects of *Sphk1* deficiency on endothelial function and vascular superoxide anion production

**Figure 5:**
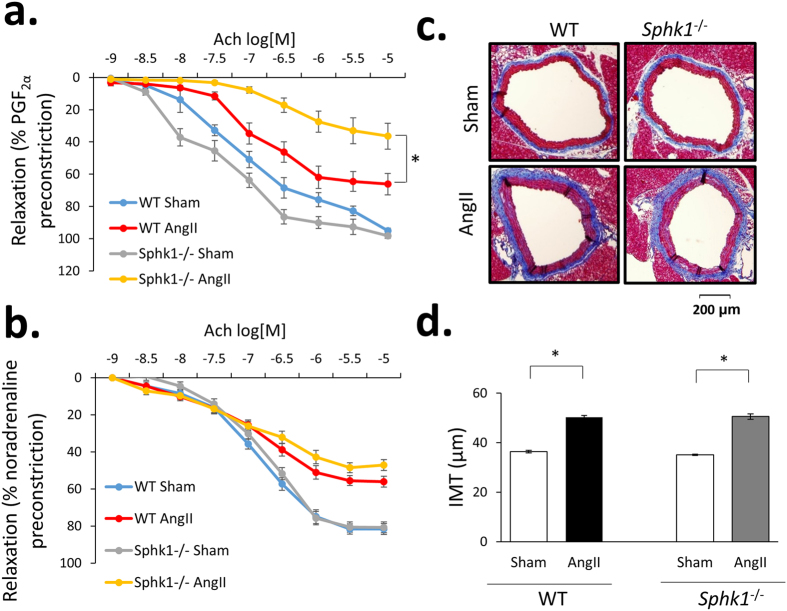
Effects of *Sphk1* deletion on endothelial function and intima-media thickness. (**a**,**b**) Tissue organ bath was performed using 2 mm aortic rings (**a**) or 2^nd^ order mesenteric arteries (**b**) from WT and *Sphk1*^−/−^ mice with an assessment of relaxation in response to increasing concentrations of acetylcholine. Vessels were precontracted using concentration of vasoconstricting agent (PGF2α or noradrenaline) required to achieve 90% of maximal contraction as determined by testing increasing doses of certain vasoconstrictor, n = 6/group; (**c**,**d**) Masson’s trichrome staining of representative aortas of WT and *Sphk1*^−/−^ mice (**c**) and comparison of mean IMT of all groups studied (**d**), n = 5 for Sham groups, n = 7 for AngII groups; *p < 0.05. WT = wild type; Ach = acetylcholine; SNP = sodium nitroprusside, [M] = molar concentration.

Lack of *Sphk1* was associated with a significant reduction of vasorelaxation in response to acetylcholine (Ach) in aortas of Ang II-infused mice (Fig. 5a) when compared to WT aortas from Ang II-infused mice. A similar trend was observed in mesenteric arteries (Fig. 5b). No differences in sodium nitroprusside (SNP)-induced vasodilation between WT and *Sphk1*^−/−^ mice were observed (Supplementary Figure 4b,c). While *Sphk1*^−/−^ was linked to concomitant enhancement of endothelial dysfunction with opposing decreased vascular contractility and moderately reduced blood pressure, it had no significant effect on intima media – thickness hypertrophy in Ang II-induced hypertension (Fig. 5c,d).

Significantly elevated *CYBB* (encoding Nox2 NADPH oxidase) expression in hypertensive *Sphk1*^−/−^ mice as compared to hypertensive WT mice, both in the aorta and mesenteric arteries, was observed (Supplementary Figure 5a). No significant changes in *Nox1* or *Nox4* expression between *Sphk1*^−/−^ and WT mice were observed (data not shown). AngII infusion significantly elevated production of superoxide anion in both *Sphk1*^−/−^ and WT mice. However, this increment was significantly higher in *Sphk1*^−/−^ mice as compared to WT mice (Supplementary Figure 5b). Similar experiments performed on aortic homogenates using fluorescent dihydroethidium (DHE) marker, showed a similar trend of association which was not significant (p = 0.22, for comparison of hypertensive *Sphk1*^−/−^ vs WT mice).

In summary, results obtained using *Sphk1*^−/−^ model and acute pharmacological modulation indicate that S1P generated via Sphk1 pathway exerts pro-contractile effects within vascular smooth muscle cells (VSMC) and an opposing protective role on the level of endothelium function.

### Serum S1P levels and vascular function in humans

Serum S1P level significantly correlated with impaired endothelial function (lower ln (Reactive Hyperemia index-RHI)) and higher aortic systolic pressure (ASP) levels, in patients with cardiovascular risk, assessed by EndoPAT and SphygmoCor respectively. At the same time, serum S1P was not significantly associated with age, sex or smoking status. No significant correlation between S1P and absolute office SBP, DBP, IMT, pulse wave velocity (PWV) or augmentation index (AIx) was observed (Table 2). Analysis of dichotomized variables showed that subjects with abnormal lnRHI (n = 20) or AIx (n = 19) level had significantly higher S1P level (respectively median of 22.3 (IQR:18.6–25.2) μM and 22.3 (IQR:16.8–25.5) μM) as compared to the rest of subjects (respectively median of 19.4 (IQR:16.2–22.6) μM and 19.5 (IQR:16.2–22.8) μM). No such association existed for dichotomized PWV level, confirming links of S1P to endothelial function rather than vascular stiffness.

## Discussion

Bioinformatical analysis of data obtained via transcriptome profiling, employed in the current study, identified *Sphk1* as one of the key modulators of Ang II-dependent vascular dysfunction *in vivo*. While numerous genes are altered during the development of Ang II-dependent hypertension, only *Sphk1, Srpx* and *Mfap4* were common for different vascular areas studied. Sphk1 was identified as a member of numerous key biological pathways significantly altered in hypertension and its expression was induced in major arteries following Ang II stimulation *in vivo*. Therefore we postulated that *Sphk1* would play a critical role in the regulation of vascular function, BP increase, and consequently cardiac hypertrophy in hypertension. Indeed, using *Sphk1*^−/−^ mice we observed that *Sphk1* is causally linked with BP elevation upon chronic Ang II infusion*. Ex vivo* and *in vivo* experiments showed that diminished vascular contractility is a possible mechanism contributing to a partial protection from Ang II-induced hypertension in mice lacking *Sphk1*. Surprisingly, the reduction of BP increase was only moderate and was not associated with a significant reduction of vascular dysfunction characterized by intima-media hypertrophy. Further studies have identified that in opposition to overall protection from hypertension and decreased vascular contractility, endothelial dysfunction was significantly aggravated in *Sphk1*^−/−^ mice. These results clearly point to the critical but very complex role of Sphk1 in the pathogenesis of Ang II-dependent hypertension and vascular dysfunction.

S1P is a lipid signaling molecule with numerous functions in vascular homeostasis, including signal transduction, mediating proliferation and migration of endothelial cells (EC), VSMCs and cardiac myocytes^21^ with a possibly clinically relevant role in human diseases such as pulmonary hypertension or abdominal aortic aneurysm^22^,^23^. It acts via 5 known receptors and S1PR1, S1PR2 and S1PR3 are predominant in the cardiovascular system^21^. *Sphk1* plays a critical role in the generation of S1P and *Sphk1*^−/−^ mice are characterized by a systemic reduction in Sphk1 activity and depletion of S1P^24^,^25^. Identification of Sphk1 as a key member of numerous pathways altered in hypertension, caused by chronic Ang II infusion, is important, as it is in line with the previous report by Wilson and colleagues who found *Sphk1*^−/−^ mice to be protected against 2-weeks model of Ang II-induced hypertension^26^, which was not confirmed in the acute model of Ang II injection^27^. In the current study, we found a somewhat smaller reduction in BP raise in *Sphk1*^−/−^ mice following chronic Ang II infusion as compared to the previous study^26^, which could be caused by a slightly lower dose of Ang II-infused.

We believe that a moderate reduction in BP raise in *Sphk1*^−/−^ mice observed by us is caused by opposing effects of S1P in the VSMCs and ECs. Previous studies have identified possible mechanisms of pro-hypertensive effects of Sphk1 including intracellular role of S1P in calcium entry via targeting store operated calcium channels^26^. Our data may support and extend these findings, since we observed both, *in vivo* and *ex vivo*, a lower contraction of vessels with lower activity of Sphk1 in response to Phe as compared to vessels with normal Sphk1 activity. This indeed suggests Sphk1-mediated Ca^2+^ handling, since receptor-mediated membrane depolarization includes engagement of voltage-dependent calcium channels and the previous study demonstrated that S1P can activate CREB transcription factor via indirect Ca^2+^ influx due to inhibition of voltage-gated K^+^ channels in VSMCs^28^. We attempted to further identify downstream molecular targets responsible for a lower contractile response in vessels with diminished Sphk1 activity. We found that blocking S1PR2 receptor lowers both aortic and mesenteric arteries contraction in response to various stimuli. Since there is an ongoing debate concerning the specificity of antagonizing S1PR2 by JTE-013, which may have off-site effects especially in micromolar concentration^29^,^30^, we addressed this issue by testing different doses of JTE-013 and found that nanomolar concentrations of this antagonist reduce vascular contraction as well. However this effect likely disappears below 10 nM concentration of JTE-013, thus there is a need to develop a specific S1PR2 antagonist, which would facilitate studies on a role of this receptor in vessel contraction. The potential role of S1pr2 in AngII-mediated impairment of vascular function is further supported by the observed induction of expression of this receptor in the aorta of WT Ang II-infused mice, which consequently may partially mediate Ang II effects *in vivo*. Interestingly, *S1pr2*^−/−^ mice have similar BP as compared to WT controls in basal conditions^31^. However, contractile responses of vessels of *S1pr2*^−/−^ mice to KCl or Phe are impaired *ex vivo* and *in vivo* stimulation of *S1pr2*^−/−^ mice with Phe unravels lower mesenteric and renal vascular resistance and a lower mean arterial pressure as compared to WT animals^31^.

However, our study reveals the complexity of the effects of Sphk1 on the regulation of endothelial function as well. While in VSMCs S1P-dependent calcium alterations may regulate contractility via store-operated Ca^2+^ channels^26^ and S1pr2^31^, in endothelium its effects may be completely different. Our findings in *in vivo* hypertension model suggest that S1P may be an important marker of endothelial dysfunction. Experiments using *Sphk1*^−/−^ mice suggest that lack of *Sphk1* and low plasma S1P level associates with impaired endothelial function. On the other hand, hypertensive WT mice display elevated S1P level, thus we tested *in vivo* effects of chronic S1P delivery. We found that elevation of S1P plasma level associates with significant impairment of endothelial function as well as with increased vessels contractility. It is of future interest to address exact mechanisms of this finding, which, among others, may include abnormal activation of S1prs in vascular media responsible for vessel contraction and internalization or degradation of S1pr1 in endothelium due to high S1P level^32^. It has been shown that genetic or pharmacological inhibition of S1pr1 or S1pr3 does not impair Ach-induced relaxation^33^,^34^. On the other hand, agonists of S1prs such as Fingolimod or S1P may display vasorelaxant properties only when S1pr1 and S1pr3 are active^33^,^34^. Previous studies showed that sphingosine kinase promotes phosphorylation and activation of endothelial nitric oxide synthase especially via S1pr1 receptor, and associated MAPK and PI3K/Akt pathways, in order to counteract Ang II effects and to mediate various processes such as angiogenesis^35^,^36^,^37^. Moreover, stimulation of S1pr1 receptor with specific S1pr1 agonist SEW2871 *in vivo* may lower BP in Ang II-induced hypertension^33^.

As recently shown by Cantalupo and colleagues, endothelial sphingolipid metabolism remains under control of Nogo-B transcription factor, which inhibits expression of serine palmitoyltransferase that participates in the synthesis of sphingolipids *in vivo*^33^. Mice lacking Nogo-B in endothelium were protected against Ang II-induced hypertension and endothelial dysfunction, which was not the case for mice lacking Nogo-B in VSMC that were only partially protected against Ang II-induced hypertension^33^. The latter effect could be explained by a reduced myogenic tone in VSMC-Nogo-B deficient mice. These various effects of inhibition of sphingolipid synthesis on vascular contractility or endothelial function can possibly explain relatively moderate, yet significant, effect of *Sphk1* deletion on blood pressure raise in Ang II-induced hypertension in the current study, which, in our opinion, deserves further investigation. Creation of VSMC and EC-specific *Sphk1*^−/−^ mice would be of high value to dissect the role of this kinase in vascular function *in vivo*.

Recent work of Tölle and co-workers demonstrated that S1P plays a role in activation of human eNOS since both S1PR1 and S1PR3 were essential for eNOS activation in human endothelial HUVEC cells^38^. However, results from clinical studies on Fingolimod show that agonism of S1P receptors transiently lowers heart rate and SBP while a chronic treatment may increase SBP by 1–3 mmHg^39^. These effects could be attributed to the agonism and functional antagonism of Fingolimod towards S1PR1^39^. To translate our findings into human population we quantified S1P level in a cohort of 82 subjects and found that S1P correlates with a worse endothelial function, as measured by lnRHI parameter. Moreover, an association of S1P level with ASP and AIx suggests that S1P may be a marker of vascular stiffening, although, no correlation was found with a PWV as the main indicator of arterial stiffness. It remains to be explored whether there is a causal relationship between S1P and the above parameters in prospective studies. Moreover, there is a need to confirm these findings in larger, prospective cohorts as well as in cohorts derived from a general population.

Our study also showed for the first time lower cardiac hypertrophy and changes in aortic superoxide anion production following Ang II infusion in *Sphk1*^−/−^ mice. Therefore it opens another possible mechanism of end-organ protection in hypertension. Reduction in cardiac hypertrophy may reflect lower systolic blood pressure but the extent of protection suggests additional mechanisms. Indeed S1P has been reported to be involved in cardiac hypertrophy and defect in models of heart failure^40^. Reactive oxygen species and especially superoxide anion are known to impair endothelial function and contribute to vascular pathology^41^. Several sources of superoxide anion in vasculature have been identified so far and include uncoupled eNOS^42^ or NADPH oxidases, among which NOX1 and NOX2 are thought to contribute to vascular pathology the most significantly^41^. Although studies linking S1P pathway with Nox2-mediated vascular redox imbalance *in vivo* are limited, it has been shown that sphingosine kinases regulate Nox2 activity via intracellular calcium store depletion and further S100A8/A9 translocation in neutrophils^43^. Our study suggests that there is a higher expression of *Nox2* NADPH oxidase in both aortic and mesenteric compartments of hypertensive *Sphk1*^−/−^ mice as compared to WT mice. This may result in abnormally elevated level of superoxide anion production in *Sphk1*^−/−^ mice in hypertension, yet our data only partially confirm this hypothesis. Since we did not find a significantly different level of superoxide anion production between hypertensive *Sphk1*^−/−^ and WT mice using DHE-based method, further work is required to determine effects of *Sphk1* deficiency on redox balance in vascular tissues.

While we have focused on *Sphk1* as a key driver of several pathways modified in Ang II-induced hypertension, our study has identified several genes and can provide a reference for future in-depth studies. The availability of expression of the whole transcriptome allowed many types of analytical strategies to be employed in order to find genes important for the development of Ang II-induced hypertension. *Sphk1* was identified via gene set and a subsequent leading edge analyses, however, this type of analysis identified other genes that possess a known, important a role in BP regulation and vascular biology. T-cadherin (*Cdh13*), which was present in the highest number of significantly Ang II-affected gene sets in the thoracic aorta, is known to serve as an adiponectin receptor providing cardioprotective functions^44^. Variations in *CDH13* locus were also associated with metabolic syndrome, adiponectin levels and BP in humans^45^,^46^. Superoxide dismutase 1 (*Sod1*), identified as a 3^rd^ top gene in our leading edge analysis, degrades superoxide anion to peroxide. *Sod1*^−/−^ mice have impaired vascular remodeling due to low NO bioavailability^47^, and SOD1 is highly expressed and functionally important in human vasculature^48^. An attempted follow-up of the loci known to associate with BP in GWAS^3^, identified subunits of soluble guanylate cyclase and *Cnnm2* among transcripts that expression was the most significantly different between hypertensive and normotensive mice. Soluble guanylate cyclase is a sensor of NO in VSMC, sensitive to Ang II stimulation, and its function in the regulation of BP is well established^49^,^50^, while Cnnm2 is relatively less studied in the context of hypertension and may be involved in maintaining Mg^2+^ balance in blood vessels^51^. Of importance variations in the coding region of *S1PR1* locus has been recently investigated in a large cohort with respect to their functional role^52^. Interestingly, one rare variant was associated with protection from coronary artery disease, which implicates that variations in the function of S1P-S1PR1 pathway may be of clinical significance.

In summary, vascular transcriptome profiling employed in the present study, allowed to identify gene causally related to the development of vascular dysfunction *in vivo*. S1P pathway stimulation in endothelium protects against vascular dysfunction, however, inhibition of S1P pathway in VSMC decreases vasoconstriction and is likely responsible for the observed lower BP in *Sphk1*^−/−^ mice following Ang II infusion, independently of endothelial function. Further studies are needed to explore the role of S1P in vascular redox balance in Ang II-induced hypertension. Modulation of the Sphk1 activity as well as targeting S1P receptors may provide a promising strategy for future studies aiming to prevent development of severe hypertension.

## Methods

### Chemicals

Ach, Phe, SNP, hydralazine hydrochloride, lucigenin, noradrenaline, and Ang II used for induction of hypertension *in vivo* were purchased in Sigma-Aldrich (Germany). Prostaglandin F2α (PGF2α), JTE-013 (S1PR2 antagonist), S1P, and Sphk1 inhibitor PF-543 were purchased in Cayman Chemical (USA).

### Experiments *in vivo*

Male C57BL/6J (n = 24) mice were used to assess the effects of chronic Ang II infusion on vascular transcriptome profile. All mice were bred in SPF animal facility. *Sphingosine kinase 1* knockout (*Sphk1*^−/−^) (stock No: 019095) and appropriate WT control mice (stock No: 005304; C57BL/6NJ) were obtained from Jackson Laboratory. Twelve-week-old mice underwent either sham or Ang II (490 ng/min/kg s.c.) treatment for 14 days, via a surgically implanted osmotic minipump (Alzet Model 2002, Alzet Corporation, CA, USA)^8^,^26^. Sham treatment involved infusion of the vehicle for Ang II. During treatment, all mice underwent non-invasive blood pressure measurement by tail-cuff plethysmography (Visitech BP 2000 BP Analysis System), following a period of training before commencement of the treatment protocol^8^. Two weeks after pump implantation mice were sacrificed and tissues, i.e. aorta, 2^nd^ and 3^rd^ order branches of superior mesenteric artery and heart were collected. Genotype of *Sphk1*^−/−^ mice was confirmed using real-time PCR and specific primer/probe set targeting *Sphk1*. To investigate the dependence of studied *Sphk1* responses to initial BP elevation, in a subset of animals hydralazine was co-administered ad libitum in drinking water at an approximate 5 mg/kg/day dose^53^. Mice received hydralazine for two weeks starting 1 day before osmotic minipump implantation. Effects of chronic S1P infusion on vascular function and BP were tested in normotensive mice receiving S1P solution in osmotic minipump for 12 days at a 300 nmol/kg/day dose. All experiments were performed according to relevant guidelines and Local Ethics Committee no. 1 in Kraków (Poland) approved the protocols employed (permissions no. 151/2012, 100/2013, 254/2015 and 157/2016).

### Transcriptome profiling

Isolated vessels were carefully cleaned of surrounding tissues and immediately placed in RNAlater solution (Ambion, USA). Two tissues of the same type and treatment status were pooled and RNA was isolated from such pools using Microarray RNEasy kit (Qiagen, USA), treated with DNAse I and quantified using Ribogreen reagent (Thermo Fisher Scientific, USA). Amplification and biotinylation of RNA were performed using Illumina Totalprep Amplification kit (Ambion, USA). The quality of RNA and cRNA was tested using Tapestation 2200 instrument (Agilent, USA), which resulted in the removal of samples not fulfilling quality criteria. Hybridization of cRNA to an Illumina WG-6 v.2.0 chip was performed according to the manufacturer protocol. Arrays were scanned on HiScan scanner (Illumina, USA). Recent GWAS that identified 29 loci associated with BP^3^ was used to check for an overlap between these loci and transcripts identified as significantly down- or upregulated in the current study. Transcriptomic data have been uploaded to the GEO server (series no. GSE75815).

### Quantification of mRNA expression

Vascular RNA from selected tissues of WT and *Sphk1*^−/−^ mice was isolated using Microarray RNEasy kit (Qiagen, USA). In an independent experiment thoracic aortas of hypertensive and normotensive WT mice were mechanically separated into 2, adventitia and intima-media, layers using surgical dissecting forceps under microscope without enzymatic treatment. Intima-media layer was then briefly perfused with 300 μl of Qiazol (Qiagen, USA) from the inside and the resulting eluate represented endothelial-enriched RNA fraction used for RNA isolation. The latter was characterized by, on average, 45x higher expression of *Platelet/endothelial cell adhesion molecule 1 (Pecam1)*, 15x higher expression of *Nitric Oxide Synthase 3 (Nos3)* and 140x lower expression of *Alpha 2 Actin (Acta2*) as compared to the remaining intima fraction. Reverse transcription was performed using 400 ng of RNA using High Capacity cDNA reverse transcription kit (Applied Biosystems, USA). Real-time PCR reactions were performed on the 7900HT instrument (Applied Biosystems, USA) using commercially available TaqMan assays for *Sphk1, Sphk2, S1pr1*, and *S1pr3.* Expression of *S1pr2* was quantified using QuantiFast Probe Assay (Qiagen, USA). Normalized microarray expression data were used to test the stability of expression of 24 commonly used candidate genes using Normfinder ver. 0.953 software (Supplementary Table 2)^54^. In this way, *TATA box binding protein (Tbp*) was selected as a constitutive gene for all real-time PCR experiments due to a high number of probes present on a chip and high stability of expression across all tissues examined.

### Western blotting

Immunoblotting was used to examine expression of Sphk1. Snap-frozen aortas, hearts and mesenteric arteries were homogenized in lysis buffer (in mmol/L: sodium pyrophosphate 50, NaF 50, NaCl 5, EDTA 5, EGTA 5, HEPES 10, Na_3_VO_4_ 2, PMSF 50, Triton 100 0.5%, and leupeptin, aprotinin and pepstatin, all 1 mg/mL). Proteins extracted from each lysate (30 μg) were separated by electrophoresis on 12% SDS polyacrylamide gel, and transferred to a nitrocellulose membrane. For immunoblotting, nonspecific binding sites were blocked with 5% Bovine Serum Albumin in Tris-buffered saline solution for 1 hour at room temperature. Membranes were then incubated with specific antibodies (anti-Sphk1 (Cell Signalling, no. 3297), anti-α-tubulin (Abcam, no. ab4074), anti-GAPDH (Abcam, no. ab8245) or anti-β-actin (A5441, Sigma-Aldrich) overnight at 4 °C (TBS-T, 5% BSA). After incubation with secondary antibodies for 1 hour at room temperature, signals were revealed with fluorescence using IRDye^®^800CW donkey anty-rabbit secondary antibodies and quantified densitometrically with Odyssey ClX Imaging system and Image Studio Lite (ver. 5.2) software.

### Vascular function studies

Vascular reactivity was measured using 750 TOBS instrument (DMT, Denmark) in isolated 2 mm segments of the aorta in organ chambers as previously described^8^. Contraction of 2 mm aortic segments in response to increasing concentrations of Phe was tested. Endothelium-dependent relaxations to Ach was measured in similar segments of the aorta in organ chambers as reported^8^. In some experiments mesenteric arteries were pre-incubated for 30 min. in KREBS buffer containing 5 μM of JTE-013 or 100 nM PF-543, while control rings were incubated with vehicle (0.1% DMSO). Experiments concerning mesenteric arteries and their contraction in response to Ang II or noradrenaline and relaxation in response to Ach were performed using 610 M myograph (DMT, Denmark).

### Vascular superoxide production

Superoxide production was measured using lucigenin-enhanced chemiluminescence (LgCL). The harvested aortic segments, stored on ice, were cut open to expose endothelial surface and equilibrated for 30 min in aerated Krebs-HEPES buffer at 37 °C. LgCL from intact vessels was measured in 2 ml buffer containing lowconcentration lucigenin (5 μM) using Berthold FB12 luminometer. Superoxide production was expressed as relative light units (RLU) per second per mg of dry weight of the vessel. An alternative, DHE-based method was used to quantify superoxide anion production in aorta homogenates of AngII-infused WT and *Sphk1*^−/−^ mice (n = 5 for both groups)^55^. Fluorescence measurements were performed in 37 °C using Synergy H4 instrument. Protein content in homogenates was normalized using Bradford reagent.

### Heart and aorta histology

Aortas and hearts were fixed using formalin solution and stored in 70% EtOH. Fixed tissues were stained using Masson’s trichrome (Sigma-Aldrich, Germany) according to the manufacturer protocol. ImageJ (ver. 1.49)^56^ was used to quantify intima-media thickness (IMT) in cross-sectional sections of aorta and area of cardiomyocytes in cross-sectional sections of hearts. We also quantified IMT of cardiac arteries of similar size in the left ventricle (diameter of approximately 100 μm; 1–2 arteries per heart) and percent of fibrotic changes in heart sections using ImageJ.

### Quantification of S1P level and Sphk activity

Mouse aortas were homogenized for 10 min. in lysis buffer with protease inhibitor cocktail in TissueLyser LT bead mill (Qiagen, USA). Homogenates were centrifuged for 10 min. (6000 g) and resulting supernatant was used to quantify sphingosine kinase activity using ATP depletion assay according to the manufacturer’s instructions (Echelon Biosciences, USA). Amount of total protein in supernatants was quantified with Bradford reagent (Sigma, Germany) and normalized. Mouse blood was collected from right ventricle using heparin as an anticoagulant. Plasma was collected after 10 min. of centrifugation at 2000 g. Level of S1P in mouse plasma, mouse aortic homogenates (prepared as described above) and human serum was quantified using competitive ELISA test according to the manufacturer instructions (Echelon Biosciences, USA).

### Patients characteristics

Eighty two patients (Supplementary Table 3) were recruited in the J. Dietl Hospital in Kraków (Poland) and E. Szczeklik Hospital in Tarnów (Poland). Inclusion criteria included age (40–80 years), at least one, documented and conventional, risk factor to develop atherosclerosis and patient’s informed consent to participate in the study. The most import exclusion criteria included: lack of patient’s consent, positive history of major cardiovascular/lung diseases (such as pulmonary hypertension, ischaemic heart disease, stroke, cancer, chronic heart failure, atrial fibrillation, COPD, bronchial asthma), positive inflammatory status (based on medical documentation or CRP level ≥10 mg/l), steroid or immunosuppressive therapy in the last 5 years, pregnancy, breastfeeding, alcoholism, menopause or post-menopausal period with use of hormonal therapy. RHI was assessed using EndoPAT device (Itamar Medical, Caesarea, Israel). RHI was natural log transformed and was analyzed as a continuous variable as well as binary variable according to the recommended cutoff point (i.e. lnRHI≥ or <0.51). AIx, PWV and ASP were assessed using SphygmoCor device (Atcor Medical Pty Ltd, Australia). PWV, AIx and ASP were analyzed as continuous variables as well as binary variables according to the cutoff points calculated by SphygmoCor software for absolute values normalized to age, sex, SBP, height and weight. Average IMT of the carotid artery was measured using ultrasound imaging. This study was conducted according to the Declaration of Helsinki and was approved by the Jagiellonian University Bioethics Committee (approval no. KBET/7/B/2014).

### Statistical analyses

Analyses of transcriptome profiles were performed in R (ver. 3.0.2) using lumi, limma and gplots packages^57^. RNA transcripts expression intensities were normalized using a robust spline normalization method. In order to find RNA transcripts globally affected by Ang II *in vivo* additional analysis comprising all samples with adjustment for tissue type was performed. Gene set enrichment analysis was performed using GSEA software (ver. 2.1.0) and Gene Ontology biological process set^58^,^59^. Subsequent leading edge analyses were performed on gene sets that were either significantly (FDR p < 0.05) enriched in hypertension (thoracic aorta) or showed normalized hypertension enrichment score >1.4 (mesenteric arteries). All remaining statistical analyses were performed in IBM SPSS statistics software (ver. 23). For comparison of the effects of Ang II on parameters in different groups of mice, we employed two-way ANOVA or Student’s t tests. Analysis of organ chamber experiments was performed using repeated measures ANOVA. Associations between S1P level and clinical parameters were tested using Mann-Whitney U test or Spearman’s correlation test. Data are presented as means ± standard error of the mean. P values < 0.05 were considered significant.

## Additional Information

**How to cite this article**: Siedlinski, M. *et al*. Vascular transcriptome profiling identifies* Sphingosine kinase 1* as a modulator of angiotensin II-induced vascular dysfunction. *Sci. Rep.* **7**, 44131; doi: 10.1038/srep44131 (2017).

**Publisher's note:** Springer Nature remains neutral with regard to jurisdictional claims in published maps and institutional affiliations.

## Supplementary Material

Supplementary Information

## Figures and Tables

**Table 1:** Ten genes present in the highest number of gene sets most significantly enriched in hypertension in thoracic aorta and mesenteric arteries and their expression fold change in hypertensive mice as compared to normotensive mice.

Thoracic aorta				Mesenteric arteries			
Gene	Number of gene sets (out of 68)	log_2_FC	Expression change–FDR adj. p value	Gene	Number of gene sets (out of 36)	log_2_FC	Expression change–FDR adj. p value
*Cdh13*	30	0.595	0.0005	*Igfbp3*	9	0.17	0.11
*Sphk1*	29	1.221	0.0003	*Eif5a*	9	0.15	0.24
*Sod1*	29	0.191	0.0391	*Inhba*	8	0.19	0.39
*Thy1*	28	0.794	0.0013	*Mast2*	8	0.17	0.07
*Tgfb1*	22	0.284	0.0248	*Cdc23*	7	0.06	0.49
*Nme2*	21	0.283	0.0092	*Ccdc88a*	7	0.10	0.28
*Col4a3*	21	0.245	0.0278	*Sphk1*	7	0.40	0.04
*Vegfa*	20	0.330	0.0594	*Apbb1*	7	0.17	0.23
*Angptl4*	19	0.402	0.0426	*Anapc5*	7	0.18	0.17
*Htatip2*	18	0.835	0.0002	*Ppp5c*	6	0.09	0.41

log_2_FC = logarithm of Fold Change (hypertensive vs. normotensive mice).

**Table 2:** Serum S1P level and its correlations with various parameters of vascular function.

	Spearman’s R coefficient	p-value
LnRHI	−0.221	**0.046**
ASP	0.214	**0.049**
DBP	0.204	0.066
SBP	0.107	0.337
Average IMT	−0.044	0.696
PWV	−0.038	0.733
AIx	0.017	0.882

RHI = Reactive Hyperemia Index, AIx = Augmentation Index, ASP = Aortic Systolic Pressure, S1P = sphingosine-1-phosphate, IMT = intima-media thickness, PWV = pulse wave velocity.
