# Supplementary material for: Vascular transcriptome profiling identifies Sphingosine kinase 1 as a modulator of angiotensin II-induced vascular dysfunction
Source: Sci Rep. 2017 Mar 9;7:44131. doi: 10.1038/srep44131 (PMC5343497; doi:10.1038/srep44131)
Supplement: Supplementary Information [file srep44131-s1.pdf]

Supplementary Information

Vascular transcriptome profiling identifies *Sphingosine kinase 1* as a modulator of angiotensin II-induced vascular dysfunction

Mateusz Siedlinski, Ryszard Nosalski, Piotr Szczepaniak, Agnieszka Ludwig-Gałęzowska, Tomasz Mikołajczyk, Magdalena Filip, Grzegorz Osmenda, Grzegorz Wilk, Michał Nowak, Paweł Wołkow, and Tomasz J. Guzik

Supplementary Table 1: Top 20 gene sets significantly enriched in hypertension in thoracic aorta and mesenteric arteries

Thoracic aorta			Mesenteric arteries		
**Gene set**	**Normalized enrichment score**	**FDR q-value**	**Gene set**	**Normalized enrichment score**	**FDR q-value**
Skeletal Development	2.058	0.011	Regulation Of Cell Migration	1.748	0.672
Angiogenesis	2.033	0.008	Viral Infectious Cycle	1.747	0.337
Vasculature Development	2.025	0.006	Regulation Of Translational Initiation	1.702	0.369
Anatomical Structure Formation	2.016	0.005	Cellular Defense Response	1.686	0.331
Cell-Cell Adhesion	1.984	0.008	Viral Reproductive Process	1.685	0.266
Organ Development	1.964	0.009	Skeletal Muscle Development	1.677	0.239
Positive Regulation Of Response To Stimulus	1.934	0.012	Mitotic Cell Cycle	1.656	0.256
Immune Response	1.929	0.011	Viral Reproduction	1.6	0.379
Multi Organism Process	1.920	0.012	Reproduction	1.549	0.528
Regulation Of Response To Stimulus	1.905	0.012	Mitosis	1.546	0.492
Organ Morphogenesis	1.889	0.014	Regulation Of Cellular Protein Metabolic Process	1.536	0.487
Tissue Development	1.888	0.013	Protein Amino Acid Phosphorylation	1.535	0.449
Organic Acid Metabolic Process	1.887	0.012	Phosphorylation	1.534	0.417
System Development	1.883	0.012	Regulation Of Protein Metabolic Process	1.512	0.464
Proteolysis	1.877	0.013	Regulation Of Phosphorylation	1.503	0.464
Carboxylic Acid Metabolic Process	1.868	0.013	M Phase Of Mitotic Cell Cycle	1.502	0.439
Ectoderm Development	1.850	0.015	Microtubule Cytoskeleton Organization And Biogenesis	1.497	0.433
Regulation Of Cell Proliferation	1.839	0.017	Regulation Of Translation	1.495	0.414
Positive Regulation Of Cell Proliferation	1.833	0.017	Regulation Of Multicellular Organismal Process	1.494	0.395
Epidermis Development	1.831	0.016	Angiogenesis	1.479	0.421

Supplementary Table 2: Expression stability of candidate constitutive genes across all 23 samples studied, calculated using NormFinder v. 0.953 software

Gene	Stability value
Abl1	0.005
Actb	0.036
B2m	0.021
Casc3	0.013
Cdkn1a	0.031
Cdkn1b	0.009
Eif2b1	0.008
Elf1	0.010
Gadd45a	0.016
Gapdh	0.011
Gapdhs	0.003
Gusb	0.019
Hmbs	0.002
Hprt1	0.017
Ipo8	0.011
Mrpl19	0.003
mtDNA_ATP6	0.010
Pes1	0.012
Pgk1	0.007
Polr2a	0.008
Pop4	0.007
Ppia	0.007
Psmc4	0.009
Pum1	0.011
Rn18s	0.030
Rpl30	0.016
Rpl37	0.006
Rplp2	0.012
Tbp	0.005
Tfrc	0.018
Top1	0.007
Ywhaz	0.009

Supplementary table 3: Clinical characteristics of enrolled patients (n=82)

Sex, n (%) males	33 (40.2)
Age, mean (SD)	57.5 (4.0)
Ex-/current smokers, n (%)	27 (32.9)/22 (26.8)
SBP in mmHg, mean (SD)	130.4 (21.2)
DBP in mmHg, mean (SD)	81.7 (10.6)
ASP in mmHg, mean (SD)	125.3 (17.5)
Serum S1P level in µM, median (IQR)	20.1 (16.4-23.4)
lnRHI index, mean (SD)	0.71 (0.27)
AIx in %, mean (SD)	33.1 (10.4)
average IMT in mm, mean (SD)	0.88 (0.13)
PWV in m/s, mean (SD)	9.0 (2.4)

RHI=Reactive Hyperemia Index, AIx=Augmentation Index, ASP= Aortic Systolic Pressure, S1P=sphingosine-1-phosphate, IMT=intima-media thickness, PWV= pulse wave velocity

Supplementary figure 1: Western Blot analysis of Sphk1 in mesenteric arteries, aortas and hearts of hypertensive and normotensive mice.

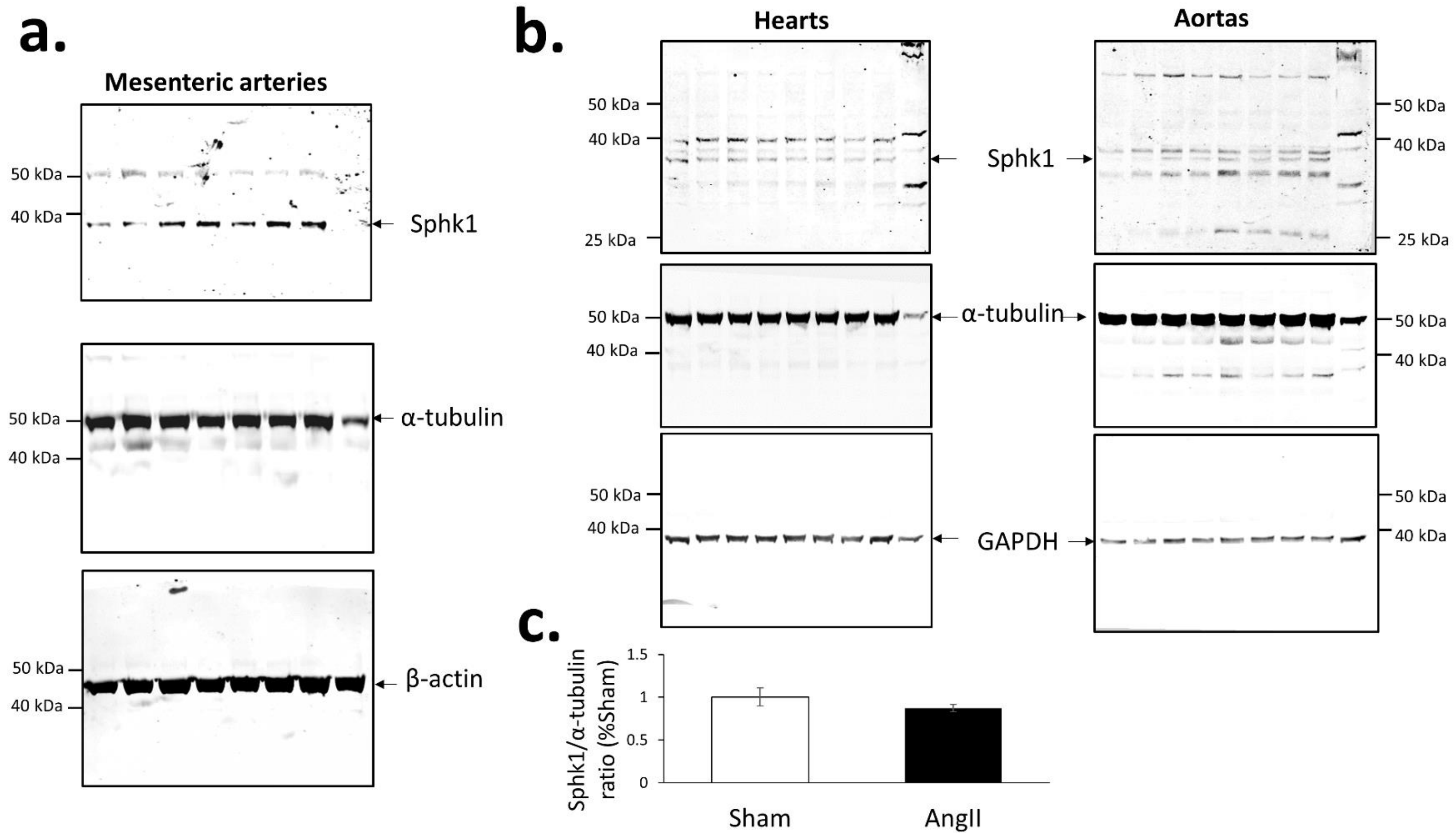

(a) Mesenteric arteries (from the left: 3x Sham, 4x AngII, 1x*Sphk1*$^{-/-}$) were used to assess the expression of Sphk1 using Western Blot method with a use of α-tubulin as a loading control. In order to confirm a specificity of the band detected and to ensure a proper loading of *Sphk1*$^{-/-}$ tissue, membrane was reprobed using antibody against β-actin. (b) In an independent experiment, heart and aortic tissues (from the left (4x Sham, 4x AngII, 1x *Sphk1*$^{-/-}$ heart) were used to assess the expression of Sphk1 using Western Blot method with a use of α-tubulin as a loading control. In order to confirm a specificity of the band detected and to ensure a proper loading of *Sphk1*$^{-/-}$ tissue, membrane was reprobed using antibody against GAPDH. In both analyses (a, b), lack of band in the *Sphk1*$^{-/-}$-derived tissue was used to identify Sphk1-specific band.
(c) Densitometric analysis of Sphk1 expression in hearts of Sham vs. AngII-infused mice (p=0.38).

Supplementary figure 2: Effects of chronic S1P infusion on vascular function *in vivo*

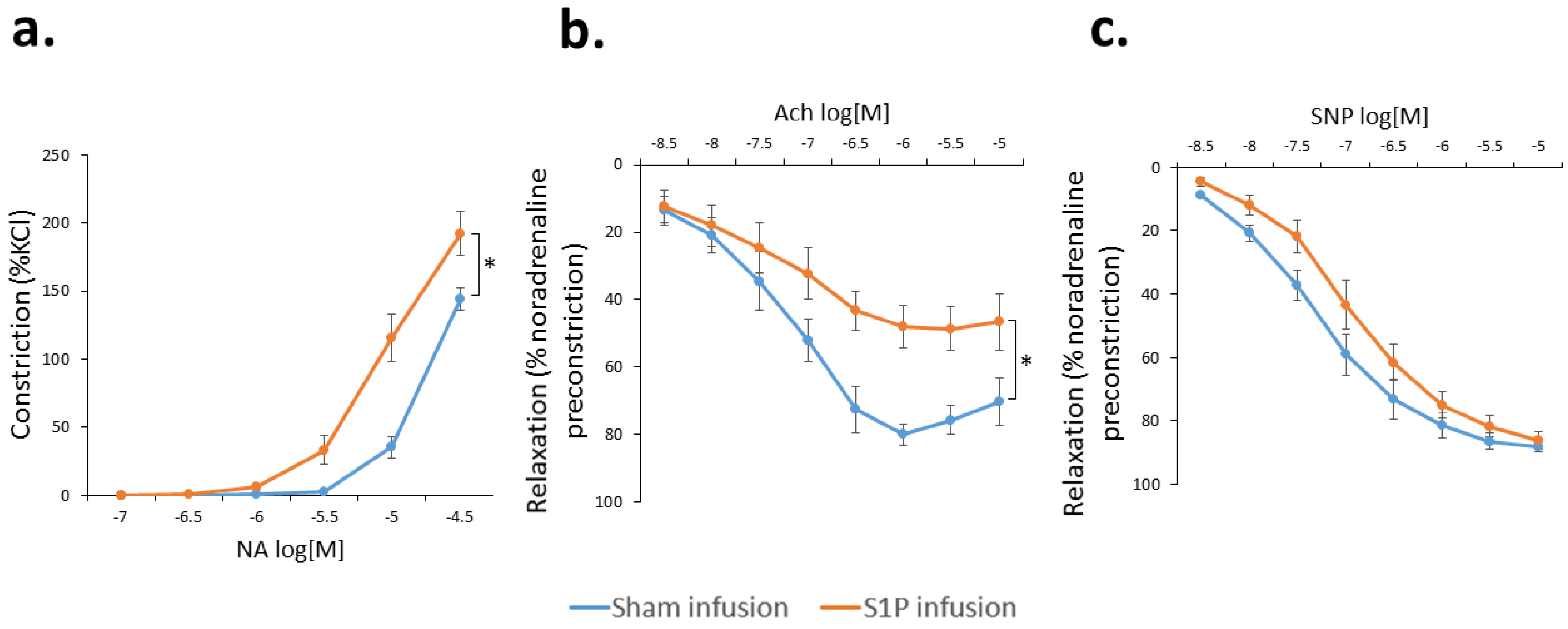

Effects of chronic S1P infusion on vascular function were tested in normotensive WT mice receiving sphingosine-1-phosphate (S1P) or Sham solution in osmotic minipump for 12 days at a 300 nmol/kg/day dose (n=5/group). Vascular function studies were performed using mesenteric arteries and 610M myograph (DMT, Denmark). Vascular contraction in response to noradrenaline (a) as well as vasorelaxation in response to Ach (b) or SNP (c) is presented. Prior vasorelaxation experiment arteries were precontracted using concentration of noradrenaline required to achieve 90% of maximal contraction as determined by testing increasing doses of this vasoconstrictor.

WT=wild type; Ach=acetylcholine; NA=noradrenaline; SNP=sodium nitroprusside, [M]=molar concentration

*$p<0.05$

Supplementary figure 3: Characteristics of hearts from WT and *Sphk1*$^{-/-}$ mice

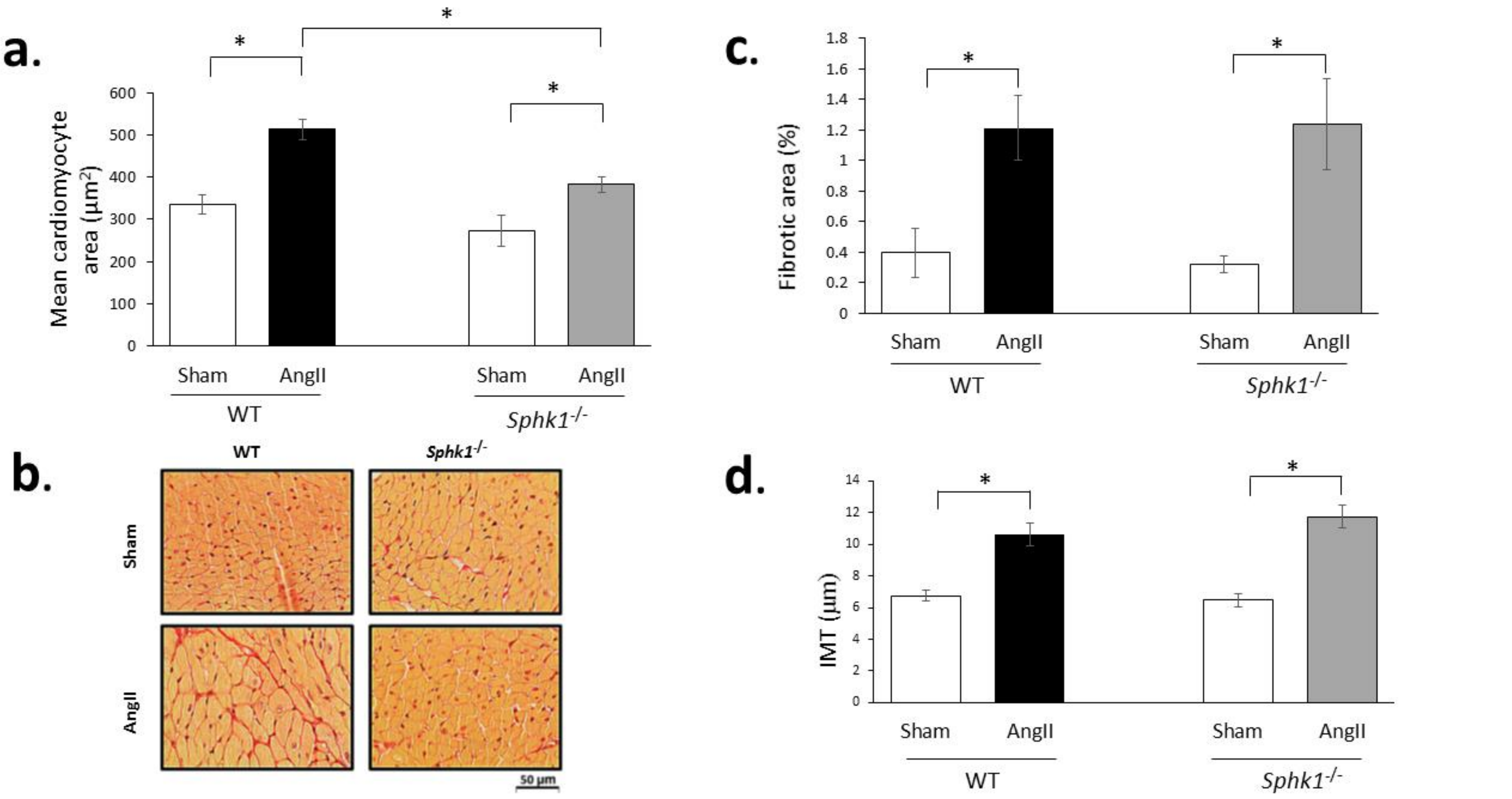

Crossectional sections of hearts (n=5-8/group) were used to quantify average cardiomyocyte size using ImageJ software (a). Representative sections are presented (b). Similarly, Masson's trichrome-stained sections were used to quantify the percentage of hearts fibrotic area using ImageJ (c). IMT of cardiac arteries (n=1-2/heart) found in the left ventricle with a diameter of approximately 100μM was quantified (n=5-8/group).

*$p<0.05$

Supplementary figure 4: SNP-induced relaxation of mesenteric arteries and thoracic aortas from WT and *Sphk1*$^{-/-}$ mice and dose-dependent impact of JTE-013 on aortic contraction *ex vivo*

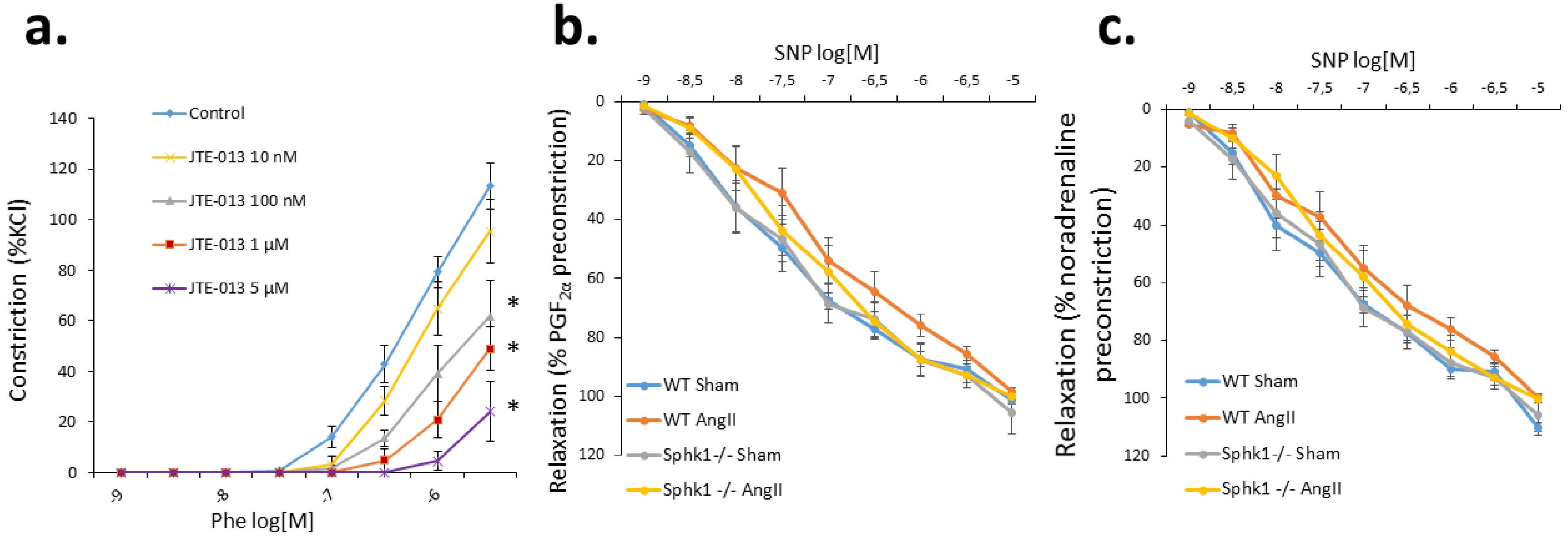

Tissue organ bath experiments were performed using 750 TOBS (a,b) or 610M (c) instruments (DMT Denmark). Phenylephrine-induced constriction of WT aortic rings preincubated in different concentration of S1PR2 antagonist JTE-013 is presented (a). SNP–induced relaxation of aortic rings (b) and mesenteric arteries (c) of hypertensive and normotensive WT and *Sphk1*$^{-/-}$ mice are shown.

SNP=sodium nitroprusside, Phe=phenylephrine; PGF2α =Prostaglandin F2α

*p<0.05 as compared to control

Supplementary figure 5: Effects of AngII infusion and *Sphk1* deletion on superoxide anion production and *CYBB* expression

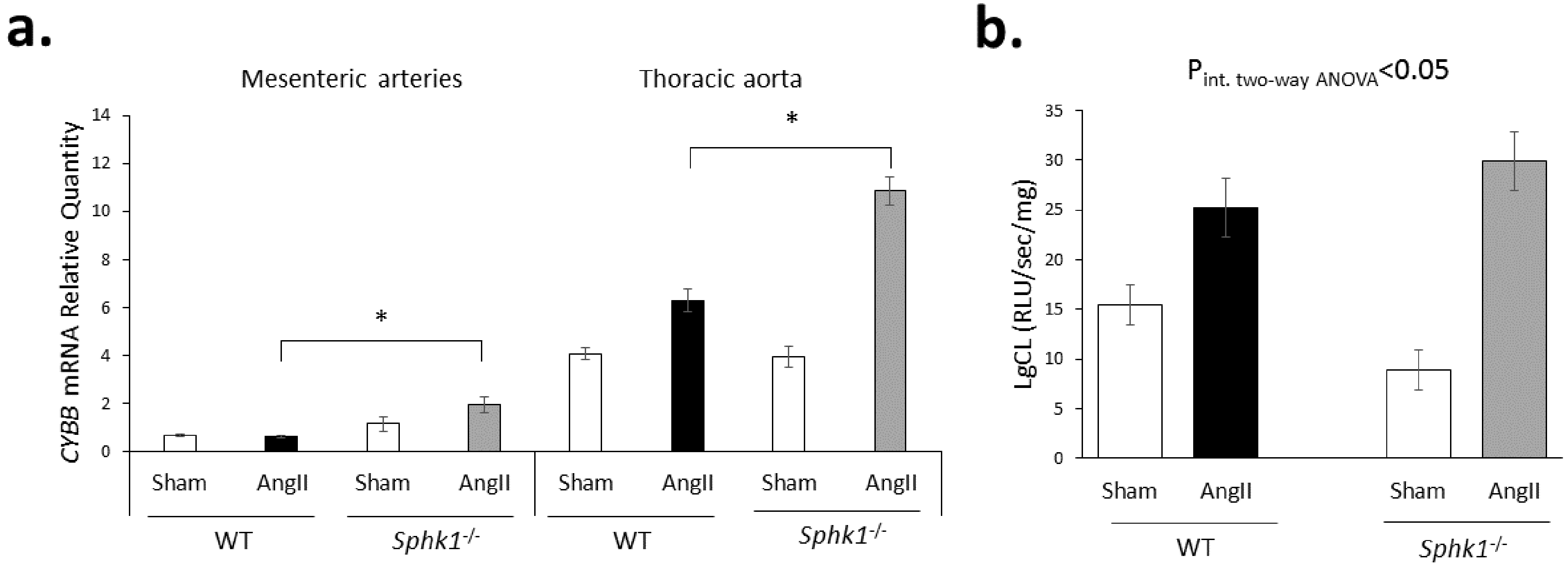

(a) Expression of *Cybb* mRNA was analyzed by real-time PCR and was normalized to the housekeeping *Tbp* gene in vascular compartments studied. *Cybb* mRNA quantity is depicted relative to the expression observed in the mesenteric arteries of Sham WT group (n=6-8/group).
(b) Aortic segments were incubated in 2 ml Krebs-HEPES buffer containing 5μM lucigenin using Berthold FB12 single tube luminometer, modified to maintain 37°C temperature. Superoxide production was quantified as relative light units (RLU) per second per mg of a dry vessel tissue, n=6/group;

*$p<0.05$

Cybb=cytochrome b-245 beta chain; Tbp= TATA box binding protein; LgCL=Lucigenin-dependent chemiluminescence
